# Personality, Executive Control, and Neurobiological Characteristics Associated with Different Forms of Risky Driving

**DOI:** 10.1371/journal.pone.0150227

**Published:** 2016-02-24

**Authors:** Thomas G. Brown, Marie Claude Ouimet, Manal Eldeb, Jacques Tremblay, Evelyn Vingilis, Louise Nadeau, Jens Pruessner, Antoine Bechara

**Affiliations:** 1 Research Centre of the Douglas Mental Health University Institute, Montreal, Quebec, Canada; 2 Department of Psychiatry, McGill University, Montreal, Quebec, Canada; 3 Foster Addiction Rehabilitation Centre, St. Philippe de Laprairie, Quebec, Canada; 4 Faculty of Medicine and Health Sciences, University of Sherbrooke, Longueuil, Quebec, Canada; 5 Department of Family Medicine and Epidemiology & Biostatistics, University of Western Ontario, London, Ontario, Canada; 6 Department of Psychology, University of Montreal, Montreal, Quebec, Canada; 7 Department of Psychology, University of Southern California, Los Angeles, California, United States of America; Technion Israel Institute of Technology, ISRAEL

## Abstract

**Background:**

Road crashes represent a huge burden on global health. Some drivers are prone to repeated episodes of risky driving (RD) and are over-represented in crashes and related morbidity. However, their characteristics are heterogeneous, hampering development of targeted intervention strategies. This study hypothesized that distinct personality, cognitive, and neurobiological processes are associated with the type of RD behaviours these drivers predominantly engage in.

**Methods:**

Four age-matched groups of adult (19–39 years) males were recruited: 1) driving while impaired recidivists (DWI, n = 36); 2) non-alcohol reckless drivers (SPEED, n = 28); 3) drivers with a mixed RD profile (MIXED, n = 27); and 4) low-risk control drivers (CTL, n = 47). Their sociodemographic, criminal history, driving behaviour (by questionnaire and simulation performance), personality (Big Five traits, impulsivity, reward sensitivity), cognitive (disinhibition, decision making, behavioural risk taking), and neurobiological (cortisol stress response) characteristics were gathered and contrasted.

**Results:**

Compared to controls, group SPEED showed greater sensation seeking, disinhibition, disadvantageous decision making, and risk taking. Group MIXED exhibited more substance misuse, and antisocial, sensation seeking and reward sensitive personality features. Group DWI showed greater disinhibition and more severe alcohol misuse, and compared to the other RD groups, the lowest level of risk taking when sober. All RD groups exhibited less cortisol increase in response to stress compared to controls.

**Discussion:**

Each RD group exhibited a distinct personality and cognitive profile, which was consistent with stimulation seeking in group SPEED, fearlessness in group MIXED, and poor behavioural regulation associated with alcohol in group DWI. As these group differences were uniformly accompanied by blunted cortisol stress responses, they may reflect the disparate behavioural consequences of dysregulation of the stress system. In sum, RD preference appears to be a useful marker for clarifying explanatory pathways to risky driving, and for research into developing more personalized prevention efforts.

## Introduction

Driving is a common activity in which one’s behaviour can have dire consequences for personal and public safety. Road traffic crashes rank first as the most important preventable contributor to global morbidity; an estimated 14,000 people per day, 5 million people per year, have their lives curtailed by injury and death [1–4]. Human factors are responsible for an estimated 90% of all crashes [5], which primarily involve engagement in risky driving (RD) behaviours such as driving under the influence of alcohol, speeding, recklessness, distracted driving, and fatigued driving. Drivers prone to repeated episodes of RD are over-represented in crashes [6–10], making them logical targets for selective prevention strategies when detected by repeated convictions [11, 12]. At the same time, the repeated offending by risky drivers (RDs) suggests that they are refractory to general rational appeals (e.g., public health messages), deterrence (e.g., police enforcement and sanctions), and remedial countermeasures (e.g., educational programs). The design of more targeted interventions could be helpful in this regard, but this will require a clear understanding of the explanatory pathways to persistent RD behaviour [1].

Two major perspectives influence the research into individual factors underlying engagement in persistent RD. One perspective akin to problem behaviour theory posits that RD reflects a propensity for engaging in multiple forms of risk taking that are inter-related by common underpinnings [13, 14]. Drivers involved in serious crashes have been observed to engage in multiple RD and related problem behaviours (e.g., substance misuse) than those who are not [6]. Moreover, compared to safe drivers, RDs are more likely to show the personality features (e.g., sensation seeking, impulsivity and reward sensitivity), and attitudes (e.g., competitiveness, defiance of authority and positive alcohol expectancies) that are postulated by problem behaviour theory [15–18]. At the same time, the members of the RD population exhibit marked heterogeneity in their individual characteristics, risk-taking preferences, and response to prevention efforts [19–23].

Alternatively, a typological perspective proposes that the RD population includes subgroups whose members share specific characteristics and unique explanatory pathways to their risk-taking [24]. Along these lines, better outcomes may result from exposing members of a subgroup to an intervention specifically designed to interrupt these pathways [25]. Several studies have relied on statistical methods such as cluster analysis to derive RD subgroups (e.g., [21, 24, 26]), but the clinical relevance of this approach is often unclear. At the same time, even obvious characteristics may be puzzling as to their explanatory role. For example, along with access to a vehicle, heavy drinking is a precondition for driving while impaired (DWI), but it is insufficient for explaining why some heavy drinkers engage in DWI while most do not [27, 28].

A potentially more clinically meaningful approach involves focussing on distinct individual risk-taking patterns. For instance, a recent study conducted in military personnel [29] investigated the association between the frequency of unintentional mistakes versus deliberate rule violations and the personality antipodes of fearlessness and fearfulness. Fearlessness was associated with more deliberate rule violations, while fearfulness was associated with unintentional mistakes. Two other related studies in general student and adult samples [30, 31] attempted to link self-reported RD, including speeding, impaired driving, unbelted driving and fatigued driving attitudes to specific personality features. Both studies uncovered relationships, but they were inconsistent between studies. Overall, these findings provide cautious support for the hypothesis that disparate RD behaviours are associated with distinct individual features. At the same time, the literature has relied predominantly on self-report data for gauging both driving behaviour and individual characteristics, which may increase the risk of bias from subjectivity and shared method variance. Moreover, the preponderance of healthy non-offender samples in the literature raises questions about the external validity of the findings to the RD groups at which selective prevention efforts should be targeted.

As an adjunct to the self-report and psychometric variables commonly used in traffic safety research, measurement of neuropsychological and neurobiological processes may provide additional insight into individual differences in risk taking. Moreover, it may provide data less susceptible to subjectivity and shared method variance [32, 33]. Imaging studies reveal that safe driving in a virtual reality environment (i.e., driving simulation) engages executive control systems associated with the prefrontal cortex, including error monitoring, inhibition, vigilance, planning and decision making [34–37]. The corollary of this observation is also observed; the cognitive processes associated with RD resemble those seen in other risk-taking behaviours [38]. For example, disadvantageous decision making and weaker inhibitory control capacities have been associated with RD in studies with different driver samples (e.g., [39–43]). At present, whether specific executive control processes are more strongly linked to certain subgroups within the RD population has not been adequately investigated.

Dysregulation of a major hormonal stress system, the hypothalamic-pituitary-adrenal (HPA) axis, is also associated with risk taking [44–48]. HPA-axis activation occurs after exposure to physiological stressors like cold and pain, but even more to psychological stress. In humans, the major hormones of the HPA axis are corticotrophin releasing factor (CRF), adrenal corticotrophin hormone (ACTH), and cortisol. Stress exposure results in limbic, cortical and other afferent inputs to trigger hypothalamus release of CRF. CRF is transported to the anterior pituitary and stimulates the release of ACTH, which in turn stimulates the synthesis and release of cortisol by the adrenal cortex to prepare the body for coping with stress. Cortisol in blood binds to brain receptors in the amygdala, prefrontal cortex and hippocampus. Hence, the cortisol stress response may demarcate individual differences in functioning associated with these areas.

Two neurobiological theories link the cortisol stress response to risk-taking behaviour [48]. Stimulation-seeking theory conjectures that chronic under-arousal is experienced as an aversive physiological state that some individuals relieve through risk taking. Alternatively, fearlessness theory suggests that under-arousal to stress and risk taking interferes with avoidance learning, thereby encouraging repeated risk taking. Previous studies have found blunted cortisol stress responses in different RD samples [49–51], however comparative examination of cortisol stress response related to different forms of RD has been lacking. In addition, which of the above theories may better explain specific forms of RD has not been systematically examined.

The present study probed the personality, cognitive and neurobiological characteristics of drivers who present with three distinct and clinically prevalent forms of RD: driving while impaired by alcohol (group DWI), non-alcohol involved reckless driving (group SPEED), and a mixed pattern involving both DWI and non-alcohol related reckless driving (group MIXED). Our exploratory hypothesis was that repeated engagement in a specific form of RD is a clinical marker of the distinct personality, cognitive, and neurobiological processes that underlie it. If this hypothesis was supported, the findings could point to how individualized intervention approaches to different forms of RD might evolve.

## Methods

### Site and participant recruitment

The Addiction Research Program of the Douglas Mental Health University Institute, a McGill University-affiliated teaching hospital, was the site of participant recruitment and testing. The Institute’s Research Ethics Board approved all study procedures (certificate #11/23). Male drivers aged 19–39 years were recruited. Group recruitment was purposefully directed to obtain four distinct age-matched groups: three RD groups (DWI, MIXED, SPEED) and a control group (CTL). Based upon the RD literature [52, 53], minimal inclusion criteria for each RD group were as follows: 1) DWI: [≥2 DWI convictions at a blood alcohol level >80 mg/100ml OR ≥1 DWI conviction at a blood alcohol level >150 mg/100ml] AND [no other non-alcohol traffic offences in the last 10 years]; 2) MIXED: [≥1 DWI convictions in the past 10 years] AND [≥1 moving traffic violations in the previous two years]; and 3) SPEED: [≥3 moving traffic violations not involving alcohol in the previous two years] AND [no DWI arrests in the past 10 years]. For inclusion into group CTL, drivers met no RD group criteria, possessed no lifetime DWI arrests or convictions, and had not lost more than 2 demerit points for any other non-criminal highway code violation in the last two years. General study exclusion criteria were: i) suffering acute or chronic ill health that precluded safe participation; ii) reading skills of less than 6th grade level determined by academic achievement; and iii) evidence (either self report or biological) of recent alcohol or drug use within 12 hours of the testing session. Recruitment relied on advertisements placed in local newspapers and on the research team’s website, with $180 CDN offered as compensation for participation.

### Tasks and questionnaires

#### Sociodemographics, substance use, driving history and criminal behavior

The Addiction Severity Index [54, 55] provided information on sample sociodemographics and family history of alcoholism. The Michigan Alcoholism Screening Test (MAST) is a 24-item questionnaire that provided an index of lifetime alcohol problem severity and related negative consequences [56]. The Alcohol Use Disorder Identification Test (AUDIT) [57] is a 10-item questionnaire that assessed alcohol misuse and its negative consequences in the previous 12 months. The Drug Abuse Screening Test (DAST) [58] is a 20-item questionnaire that provided an index of lifetime drug problem severity [59]. The Timeline Follow Back (TLFB) [60] involved presentation of a calendar to aid recall and measurement of daily alcohol and drug use in the past 90 days, specifically the frequency of days when five or more standard drinks or any drugs were consumed. A Breathalyzer® was used to objectively detect current blood alcohol concentration, and Drugwipe® 6S was used to detect recent (previous 12 hours) cannabis, cocaine and benzodiazepine use—the most common drugs detected in DWI offenders [61]. For driving history, participants were queried about their age of licensing, estimated annual kilometers driven, and involvement in serious crashes involving either ≥ $1500 CDN of damage or injuries over the last five years. The legal section of the Addiction Severity Index documented self-reported convictions for major driving violations (e.g., speeding, reckless driving, running stoplights, impaired driving, etc.), as well as lifetime frequency of non-driving criminal convictions.

#### Risky driving behavior

The Manchester Driving Behaviour Questionnaire is a 24-item self-report instrument that measured engagement in four behaviours related to crash risk: ordinary violations, aggressive violations, lapses, and errors [62–64]. Real-time measurement of driving behaviour was undertaken via a portable driving simulator developed by co-author MCO and colleagues at the University of Sherbrooke. Driving simulation has been shown to possess both ecological and convergent validity in relation to normal driving performance (e.g., [65–67]). The simulator was composed of a CPU, three screens, and a steering wheel, accelerator and brake pedals that enabled drivers to interact with realistic driving scenarios. After a 10-minute practice session, participants undertook a 12.5 km simulated drive that included a highway section with a 100-km/h speed limit and merging ramps at 70 km/h, and an urban section with common driving challenges including traffic, turns at intersections, pedestrians, and construction sites. Participants were instructed to drive as they normally would. Driving measures that could indicate RD were: mean speed in highway settings sampled several times per second, waiting time in minutes before committing a risky illegal manoeuvre (crossing a solid road line) to pass a stalled vehicle at an intersection with traffic lights, and position of the acceleration pedal (range from 0 to 1 at maximum acceleration) when encountering a vehicle merging onto the roadway at matched speed.

#### Personality

The short version of the NEO Personality Inventory [68], the NEO Five-Factor Inventory (NEO-FFI) [69], measured the “Big Five” personality dimensions: neuroticism, extraversion, openness, agreeableness, and conscientiousness. The UPPS-P Impulsive Behavior Scale [70] measured five facets of impulsivity including negative and positive urgency (emotion-based impulsivity), lack of premeditation, lack of perseverance, and sensation seeking. The Sensitivity to Punishment and Sensitivity to Reward Questionnaire (SPSRQ) is based upon Gray’s model of personality [71]. Higher scores on the UPPS-P and SPSRQ connote greater impulsivity and sensitivity to reward and punishment, respectively.

#### Executive control

To measure functional executive control, the computer-assisted version of the Connor’s Continuous Performance Test (CPT-II) [72] (Multi-Health Systems) was used. Single letters appeared at three different time rates: 1/1s (seconds), 1/2s and 1/4s. Participants were asked to make a mouse click in response to every signal except the target signal (X). Three measures sensitive to executive control were used: number of commission errors and preservation errors (high scores indicate greater disinhibition), and hit reaction time (lower scores indicate greater impulsivity) [73].

The computerized BIOPAC™ version of the Iowa Gambling Task (IGT) [74] was used to measure decision-making capacities. Participants were instructed to select cards from one of four 40-card decks labeled A, B, C, and D to accumulate as much play money as possible within 100 trials. Unbeknownst to participants, the decks differed on the amount of potential gain versus the amount of potential losses. Decks A and B were set so penalties outweigh rewards, making these decks disadvantageous; decks C and D were set so gains outweigh penalties, making them advantageous. Two decision-making measures were calculated: i) decision making under ambiguity, when outcome probabilities are unknown using the average of earlier trials; and ii) decision making under risk, when outcome probabilities are known using the average of later trials [75]. Lower scores on these measures indicate more disadvantageous decision-making performance.

The Stoplight Task [76] is a risk-taking task in which participants make decisions about whether to stop at a traffic intersection, or choose to cross the intersection and risk a collision with another vehicle. Each of 40 trials presents an intersection with either a green (safe), yellow (risk of collision), or red (sure collision) light. When encountering yellow lights, participants press keyboard key to make either a safe decision to stop and wait for green, or a risky decision to go through and risk a collision. Rapid task completion and/or fewer collisions result in a token monetary reward, while caution, time delay, and/or accidents engender monetary penalties. Risk taking is operationalized as the frequency of risky decisions made. Differences in individuals’ reward- or thrill-seeking biases have an especially strong influence on task performance [77].

#### Cortisol stress response

Cortisol saliva sampling using Salivettes® (Sarstedt, St. Laurent, Quebec, Canada) occurred six times at 15 minutes intervals, three baseline intervals prior to stress task exposure and three intervals after stress task exposure. Immediately following collection of the third baseline interval, participants underwent a standardized mental arithmetic task under pressure of time and potential performance-based rewards, a protocol that reliably elicits a stress response. Peak cortisol response typically occurs at interval 5, 30 minutes after stress task exposure [49, 78]. The cortisol content of saliva (μg/100mL) was measured using the AMERLEX® Cortisol radioimmunoassay kit (cat. # 8758401; Ortho-Clinical Diagnostics, Inc. Rochester New York). Cortisol stress response was operationalized as the area under the curve with respect to increase [49, 78], namely change in salivary cortisol level from baseline (interval 3) to peak cortisol level following stress task exposure (interval 5).

## Procedures

When study candidates called the study recruiter, they were provided study information, had their questions answered, and if appropriate, were asked inclusion/exclusion questions. If they met inclusion criteria and agreed to participate, they were scheduled for an experimental session starting at 8:30 AM, and provided instructions regarding pre-session drinking, drug and cigarette use, and food and caffeine intake. Driving status information, which is in the public domain, was obtained from Quebec’s licensing authority prior to the testing session. On arrival, prospective participants were asked to present picture identification and driver licence. They were then given Informed Consent forms to read, question, and then sign if acceptable. Participants then were administered an alcohol Breathalyzer® and DrugWipe®, which if positive would result in the session being rescheduled. The first component of the assessment lasted until approximately 11:30 AM and involved health and drug screening and psychological and psychosocial assessment, interspersed with scheduled rest breaks. Following a light standard lunch, the cortisol stress task protocol lasted from 12:45 PM until 3:00 PM. Finally, participants were administered the IGT and driving simulation task to finish at approximately 4:15 PM.

### Preliminary data treatment and main analytic plan

Initial within-group diagnostic analyses of continuous variables screened for outliers. This was operationalized as a variable score that was found to be ≥ +3.3 or ≤ -3.3 SD from the group mean; when detected, outliers were transformed to next extreme score plus one unit [79]. The frequency of outliers for any given variable ranged from 0 to four, with specific details concerning outliers for each variable reported in the Results section. Missing data on some variables, being infrequent (i.e., ≤ 3 cases or ≤ 2.2%) and apparently random, were not replaced and hence were omitted from analyses of that specific variable. Details concerning missing data for each analysis are reported in the Results section.

Descriptive between-group comparisons on sociodemographics, self-reported driving history and behaviour, substance use, and driving simulation performance used ANOVA followed by Bonferroni *post hoc* analyses. Effect sizes for significant group contrasts are reported as partial eta squared (η^2^). Robust or non-parametric statistics were used to confirm the analyses above with data exhibiting severe, non-correctable non-normal distributions and/or variance heterogeneity, and are reported in Results when used. In order to meaningfully test our hypothesis (i.e., to characterize RD groups on personality, cognitive, and neurobiological variables), we adopted an *a priori* statistical strategy. Three planned orthogonal contrasts for each variable was undertaken, with group CTL used as the reference against which each RD group was contrasted. Overall alpha was set to p ≤ 0.05 (two-tailed) for each set of contrasts, with significant differences from group CTL indicated in the relevant table by confidence intervals not intersecting 0. Effect sizes for significant group contrasts are reported in the Results section as partial η^2^ for the group main effect based upon a preliminary ANOVA.

## Results

### Recruitment and sociodemographics

Prior to the experimental session, four participants tested positive for drugs. One was rescheduled, but subsequently lost to attrition, and another excluded himself from the protocol. The final two remained eligible and proceeded; in one case, drug use was considered to have occurred prior to the standard 12-hour pre-session abstinence duration, and in the other case, the drug test was judged to represent a false positive. In total, 138 male drivers were recruited: group CTL, n = 47; group DWI, n = 36; group Mixed, n = 27; and group Speed, n = 28.

Table 1 summarizes the characteristics of groups on sociodemographic variables, as well as the results of descriptive comparisons. Group differences were detected on ethnic composition (i.e., percent White vs. non-White including Black, First Nations, Asian, Hispanic or other), χ^2^(3) = 12.5, p = .006. Hence, to adjust for the potential influence of this factor on the findings (e.g., [80]), we entered ethnic composition as a covariate in analyses of substance use, parametric and simulated driving variables, and personality, cognitive, risk taking and neurobiological measures, both as a main and interaction (i.e., with group) effect.

**Table 1:** Sociodemographics, substance use, and driving and lifetime criminal history of the control group (CTL; n = 47), driving while impaired group (DWI; n = 36), mixed group (MIXED; n = 27), and non-alcohol reckless driving group (SPEED; n = 28), and between-group comparisons.

	CTL	DWI	MIXED	SPEED	p^c^
	*M (SD)*	*M (SD)*	*M (SD)*	*M (SD)*	
**Sociodemographics**					
Age	30.1 (6.2)	30 (5.7)	27.8 (6.1)	28.7 (5.0)	
Ethnicity					**^d^
% White	70.2	88.9^4^	85.2	53.6	
% Non-White^a^	29.8	11.1	14.8	46.4	
Highest level of education					
% From any grade to secondary school	10.6	19.4	37.0	25.0	
% Some college or vocational training	61.7	63.9	51.9	42.9	
% Bachelor or Master degree	27.7	16.7	11.1	32.1	
Family status					
% Single, separated, divorced	70.2	69.4	81.5	67.9	
% Married/ living with a partner	29.8	30.6	18.5	32.1	
Income from all sources (last year)					
% 0—≤ 19 999 $	25.5	22.2	40.7	21.4	
% 20 000–39 999 $	34.0	33.3	25.9	46.4	
% ≥ 40 000 $	40.4	44.4	33.3	32.1	
Occupation (last 3 years)					
% Full-time or stable part-time job	76.6	63.9	55.6	78.6	
% Student or other^b^	23.4	36.1	44.4	21.4	
**Substance use**					
MAST	2.4 (2.4)	22.0(17.9)^1,3,4^	11.9 (8.8)^1^	4.4 (4.5)	***
AUDIT	4.3 (3.8)	9.9 (7.7)^1^	7.4 (4.6)^1^	6.8 (6.5)	***
DAST	0.7(1.6)	1.3(1.5)	2.4(4.1)^1^	1.8(2.3)	*
TLFB Days of ≥ 5 standard drinks in past 90 days	3.1 (5.5)	7.2 (8.4)	4.6 (5.4)	5.6 (8.7)	
TLFB Days of drug use in past 90 days	5.5 (18.8)	4.8 (15.3)	10.0 (22.9)	12.4 (24.7)	
Age at first alcohol use	14.3 (5.4)	13.8 (3.5)	14.7 (2.1)	13.5 (4.6)	
% Family history of alcoholism	27.7	47.2	27.0	25.9	
Number of cigarettes smoked/day	1.0 (2.9)	5.9 (8.2)^1^	4.4 (6.7)	3.0 (5.8)	*
**Driving history & criminal behaviour**					
Age of licensing	18.3 (3.0)	17.4 (1.6)	17.9 (2.4)	18.4 (2.3)	
Kilometers (1000’s) driven (past 5 years)	92.7 (81.8)	74.9 (123.1)	101.7 (126.2)	142.1(102.1)	
Frequency of crashes causing ≥$1500 damage or injury (past 5 years)	0.2 (0.6)	0.5 (0.7)	0.7 (0.7)	1.0 (1.6)	
Frequency of major driving violations (lifetime)	1.2 (1.6)	5.3 (7.4)^1^	7.1 (6.7)^1^	10.7 (7.4)^1^	***
Frequency of DWI convictions (lifetime)	0.0 (0.0)	1.9 (1.1)^1,3,4^	1.1 (0.4)^1,4^	0.0 (0.0)	***
Frequency of self-reported DWI episodes (past year)	0.2 (0.8)	0.3 (0.2)	0.2 (0.5)	0.5 (0.9)	
Frequency of non-driving criminal convictions (lifetime)	0.2 (0.7)	1.6 (2.9)	2.3 (4.3)^1^	1.4 (3.8)	*

Notes

^a^ Non-White includes Black, First Nations, Asian, Hispanic, and others

^b^ Other included retiree, disabled, living in institutional settings or unstable conditions, seasonal worker, unemployment or welfare, homemaker

^c^ Group differences were detected by ANOVA for continuous variables and χ^2^ for categorical data

^d^ All continuous measures of substance use, driving history characteristics, and non-driving criminal convictions were controlled for ethnicity.

* p ≤ .05

** p ≤ .01

*** p < .005.

Numerical superscripts indicate significantly higher scores than the group denoted (CTL = 1, DWI = 2, MIXED = 3, SPEED = 4).

**Abbreviations:** AUDIT: Alcohol Use Disorder Identification Test; DAST: Drug Abuse Screening Test; MAST: Michigan Alcohol Screening Test; TLFB: Timeline Followback.

### Substance use

Table 1 summarizes the substance use data, as well as the results of descriptive comparisons. The following substance use variables had outliers (frequency in brackets) that were transformed prior to analyses: MAST (2); DAST (2); TLFB alcohol (1) and drugs (2); and daily cigarettes (3). A group effect was found on the MAST, F(3,130) = 18.3, p < .001, η^2^ = .30, with *post hoc* tests identifying greater lifetime alcohol problem severity in groups DWI and MIXED versus group CTL, and greater severity in group DWI versus either group MIXED or SPEED. A group effect was also detected on the AUDIT, F(3,130) = 4.0. p < .01, η^2^ = .08, with *post hoc* tests indicating greater alcohol misuse severity in groups DWI and MIXED compared to group CTL. For drug use, a group effect on the DAST was detected, F(3,130) = 3.2, p = .03, η^2^ = .03, with *post hoc* tests identifying greater drug misuse severity in groups CTL and MIXED compared to group Control. Number of daily cigarettes consumed also differentiated between group, F(3,130) = 5.2, p = .002, η^2^ = .11, with *post hoc* tests revealing that group DWI consumed more cigarettes than group CTL.

### Driving history and criminal behaviour

Table 1 summarizes the driving history and criminal behaviour data, as well as the results of descriptive comparisons. On frequency of lifetime major driving violations, and after transformation of two outliers, a group effect was found F(3,130) = 23.2, p < .001, η^2^ = .21. *Post hoc* tests indicated more frequent major driving violations by all RD subgroups compared to group CTL, and more frequent violations by group SPEED compared to group DWI. A group effect for lifetime frequency of DWI offences was also detected, χ^2^(3) = 129.0, p < .001, with *post hoc* tests indicating that group DWI had more convictions than all other groups, and group MIXED had more convictions than either group CTL or group SPEED. After transformation of three outliers and omission from analysis of three cases due to missing data (i.e., two in DWI, one in SPEED), a group effect on the frequency of lifetime non-driving related criminal convictions was found, F(3,127) = 3.3, p = .02, η^2^ = .07, with *post hoc* tests indicating more frequent convictions in group MIXED compared to groups CTL.

### Risky driving

Table 2 summarizes self-reported driving behaviour, driving performance in simulation, and results from between-group ANOVA. On self-report measures, analyses revealed a group effect on the ordinary violations subscale of the Manchester Driving Behavior Questionnaire, F(3,130) = 3.3, p = .02, η^2^ = .07, with *post hoc* tests indicating that group SPEED scored significantly higher than either group CTL or group DWI. In driving simulation, with three cases (group MIXED) lost because of data damage, a group effect was detected on mean highway speed, F(3,127) = 6.4, p < .001, η^2^ = .13, with *post hoc* tests revealing group SPEED driving significantly faster than group CTL. After transformation of one outlier, a significant group difference was also found for accelerating to pass a merging vehicle, F(3,127) = 5.5 p < .001, η^2^ = .12. *Post hoc* tests showed that groups MIXED and SPEED were more prone to accelerate in such situations compared to either group CTL or group DWI.

**Table 2:** Self-reported risky driving and simulated driving behaviours of the control group (CTL), driving while impaired group (DWI), mixed group (MIXED), and non-alcohol reckless driving group (SPEED), and between-group comparisons.

Measures	CTL	DWI	MIXED	SPEED	
	M (SD)	M (SD)	M (SD)	M (SD)	p
**Manchester Driving Behaviour Questionnaire**					
Aggressive violations	0.9 (0.8)	0.7 (0.6)	1.0 (0.8)	1.2 (1.0)	
Ordinary violations	0.9 (0.7)	0.9 (0.6)	1.1 (0.7)	1.6 (0.9)^1,2^	*
Errors	0.4 (0.4)	0.3 (0.3)	0.5 (0.4)	0.5 (0.4)	
Lapses	0.8 (0.6)	0.8 (0.5)	0.7 (0.5)	1.0 (0.7)	
**Driving simulation**					
Mean highway speed (km/h)	76.2 (7.6)	81.3 (8.8)	81.6 (8.2)	85.4 (12.0)^1^	**
Waiting time behind stalled car (in minutes)	1.5 (0.4)	1.5 (0.4)	1.5 (0.4)	1.2 (0.5)	
Position of the acceleration pedal during interaction with merging car	0.31 (0.13)	0.34 (0.15)	0.42 (0.15)^1,2^	0.42 (0.20)^1.2^	**

Notes

* p ≤ .05

** p < .005.

Superscripts indicate significantly higher scores than groups designated (CTL = 1, DWI = 2).

### Personality

Table 3 summarizes planned comparison results for personality measures. On the Neo-FFI agreeableness scale, group MIXED scored significantly lower than group CTL, η^2^ = .08. On UPPS-P sensation seeking, groups MIXED and SPEED both scored significantly higher than group CTL, η^2^ = .08. On the SPSRQ sensitivity to reward subscale, group MIXED scored significantly higher than group CTL, η^2^ = .09. No other differences were detected.

**Table 3:** Personality, executive control, risk-taking, and cortisol stress response measures of the control group (CTL), driving while impaired group (DWI), mixed (MIXED), and non-alcohol reckless driver group (SPEED), and contrasts between CTL and risky driving groups.

Measures	CTL	DWI	MIXED	SPEED	95% CI vs. CTL		
	*M (SD)*	*M (SD)*	*M (SD)*	*M (SD)*	*DWI*	*MIXED*	*SPEED*
**NEO-FFI**							
Neuroticism	48.1 (11.9)	49.3 (10.4)	49.9 (12.2)	50.0 (11.5)			
Extraversion	57.2 (10)	58.4 (7.4)	59.5 (12.1)	57.0 (11.0)			
Openness	55.3 (10.5)	54.6 (9.2)	55.7 (10.8)	55.5 (7.4)			
Agreeableness	49.5 (8.7)	49.5 (9.4)	43.1 (10.1)	43.0 (9.6)		[-11.8, -1.7]	
Conscientiousness	54.0 (12.1)	55.3 (8.6)	52.1 (10.7)	51.3 (12.0)			
**UPPS-P**							
Lack of premeditation	18.9 (4.9)	19.9 (4.1)	21.1 (4.1)	20.3 (5.8)			
Urgency	25.2 (6.7)	25.5 (6.3)	28.0 (7.5)	29.3 (6.1)			
Sensation seeking	35.0 (7.1)	35.6 (6.3)	39.3 (5.7)	38.2 (6.1)		[0.3, 1.1]	[0.1, 8.0]
Lack of perseverance	17.2 (5.2)	15.7 (3.4)	17.0 (4.0)	17.8 (4.7)			
**SPSRQ**							
Sensitivity to Punishment	5.1 (4.1)	4.9 (3.5)	5.1 (4.1)	4.6 (3.6)			
Sensitivity to Reward	7.3 (3.5)	7.9 (3.1)	10.1 (3.4)	9.2 (2.5)		[1.0, 4.3]	
**CPT-2**							
Commission errors	49.4 (8.4)	54.3 (8.3)	52.4 (9.1)	54.5 (8.6)			[2.7,13.1]
Hit reaction time	46.3 (10.0)	41.1 (9.7)	44.0 (9.6)	42.2 (8.8)	[-10.3, -0.02]		
Perseveration errors	59.4 (56.4)	63.9 (54.5)	59.0 (27.2)	56.0 (22.8)			
**Iowa Gambling Task**							
Under ambiguity	2.3 (6.0)	2.5 (7.5)	0.41 (8.6)	-2.7 (9.3)			[-10.4, -0.8]
Under risk	8.0 (10.5)	8.4 (10.1)	4.2 (11.3)	9.8 (9.4)			
**Stoplight Task**	.29 (.14)	.33 (.12)	.34 (.19)	.40 (.16)			[0.01, 0.2]
**Cortisol**							
Mean baseline	.011 (.048)	.100 (.050)	.101 (.030)	.089 (.036)			
Stress response	.062 (.085)	.034 (.053)	.025 (.048)	.045 (.070)	[-.077, -.010]	[-.090, -.017]	[-.086, -.002]

Notes: All analyses were controlled for ethnicity. Abbreviations: CI: confidence interval. NEO-FFI: Short version of the NEO Personality Inventory; UPPS-P: Urgency, Premeditation, Perseveration, Sensation Seeking Scale; SPSRQ: Sensitivity to Punishment and Sensitivity to Reward Questionnaire; CPT-2: Continuous Performance Test version 2. Cortisol measures are reported as μg/100mL of saliva.

### Executive control

Table 3 summarizes the planned comparison results for executive control measures. Compared to group CTL, Group SPEED committed significantly more CPT-2 commission errors, η^2^ = .07, and exhibited less advantageous decision making under ambiguity on the IGT, η^2^ = .06. In addition, compared to group CTL, group DWI showed shorter hit reaction time, η^2^ = .03. On the Stoplight Task, compared to group CTL, group SPEED demonstrated significantly more risk taking, η^2^ = .06.

### Cortisol stress response

Mean baseline cortisol levels and cortisol stress responses are summarized in Table 3. Preliminary analyses were carried out to detect potential group differences on baseline measures. Repeated measures ANCOVA of the three baseline cortisol intervals, with group the between factor, and ethnic background the covariate, did not detect a significant effect of group nor a group by time interaction. A significant time effect was detected however, Greenhouse-Geisser F(1.5, 256) = 11.3, p < .001, η^2^ = .08, indicating differences between the three baseline measures irrespective of group. *Post hoc* tests indicated that both intervals 2 and 3 were lower than interval 1.

Preliminary diagnostic analyses on cortisol stress response revealed one outlier (DWI), which was transformed. Contrasting groups on cortisol stress response revealed that all three RD groups showed lower cortisol stress response than group CTL, η^2^ = .08. As this effect was common across all RD groups, exploratory ANOVA examined whether differences between RD groups could be detected, but this was not case at p ≤ .05. Finally, acute nicotine intake may influence cortisol levels [49]. Thus, sensitivity analysis retested the above contrasts after covarying the effects of average number of cigarettes smoked per day and their interactions with group. The results showed that the significant contrast between groups CTL and MIXED was maintained at p ≤ .05.

## Discussion

This study contrasted the multidimensional correlates of a low-risk control group with three age-matched groups of drivers with distinct RD patterns: DWI recidivists, drivers who repeatedly engage in non-alcohol related forms of RD, and drivers who engage in a mixed pattern involving both alcohol and non-alcohol-related RD. Preliminary descriptive analyses examined their psychosocial, substance use and driving characteristics. Between-group ethnic differences were observed, which were statistically accounted for in subsequent analyses. Greater alcohol misuse was found in groups DWI and MIXED compared to group SPEED and controls, but with group DWI showing the greatest misuse severity. This finding makes intuitive sense, given the role of alcohol misuse as a necessary precondition for DWI behaviour. Group DWI’s greater cigarette use compared to the other groups is also plausibly attributable to its frequent coupling with alcohol use. On self-reported driving history, while all RD groups reported some indices of elevated RD behaviour compared to controls, group SPEED were notably the riskiest. This finding was more directly tested and corroborated in driving simulation. Finally, group MIXED, possibly reflecting their more generalized risk-taking profile, also showed more criminal involvement compared to the other RD groups. In sum, these results support our recruitment strategy for sampling RD subgroups that engage in common, yet distinct RD patterns.

The main hypothesis tested here was that drivers who exhibit different patterns of persistent RD behaviour would also show a distinct pattern of personality, cognitive and neurobiological features. The results leaned in support of this contention with one notable exception: all RD groups exhibited significant blunting in their cortisol stress response compared to controls. As such, this result is in line with our findings from previous separate studies with different RD groups [49, 50, 51, 61], thereby pointing to dysregulation of the cortisol stress response as a non-specific neurobiological marker of RD behaviour. At the same time, the differences in the characteristics of the RD groups examined here extends this finding by suggesting that the propensity for engaging in specific patterns of RD may reflect the disparate behavioural consequences of dysregulation of the stress system.

Groups DWI and MIXED, while sharing both elevated alcohol misuse and blunted cortisol stress response compared to controls, were distinct in their personality and cognitive characteristics. The only other feature that differentiated group DWI from controls was greater impulsivity, indicated by shorter reaction time on the CPT-2. Blunted cortisol stress response accompanied by impulsivity has been frequently observed to accompany alcohol use disorder, with the former posited to represent a marker of impaired self-regulatory capacities, inherited risk for alcohol use disorder, and treatment refractoriness [49, 81, 82]. Hence, in drivers with this more severe form of alcohol misuse, frequent and unplanned episodes of heavy alcohol intake, and the acute impairments in psychomotor and cognitive capacities that ensue, represent a straightforward conduit to DWI behaviour.

At the same time, most DWI offenders do not meet criteria for a diagnosis of alcohol use disorder [83], and most heavy drinkers do not engage in DWI [27]. Moreover, the present results indicated that group DWI exhibited few other indices of behavioural risk taking when not under the influence of alcohol. Hence, more complex relationships between alcohol misuse and the propensity for DWI behaviour may be involved for many offenders. One possibility in line with the present results is that once heavy drinking has occurred, more impulsive drivers are more vulnerable to alcohol’s disruptive effects on the behavioural control mechanisms required to avoid DWI [27].

Another possibility involves dysregulation of limbic-related neural systems associated with emotional memory. In past work, we found an inverse relationship between memory capacity and frequency of past DWI convictions in sober DWI offenders [84]. Information that is emotionally charged and accompanied by elevated cortisol levels enhances memory through interactions between amygdala activity and stress hormones (e.g., epinephrine, corticosteroids) [85]. Conversely, blockade of adrenergic activity disrupts memory formation [86]. Hence, dysfunction of a medial temporal system that includes the amygdala and adjacent hippocampal structures may hinder memory formation of negative emotional events that are required for adaptive inhibition and avoidance behaviour [87]. Therefore, along with alcohol’s acute, generally negative impact on memory encoding [88], this effect could further contribute to the failure of common deterrence approaches aimed at drivers following a DWI conviction (e.g., severe financial and legal penalties) to prevent later recidivism. More direct experimental examination of these putative interactional pathways to DWI behaviour is clearly needed.

In contrast to group DWI, group MIXED exhibited blunted cortisol stress response accompanied by several risky personality and behavioural attributes. These involved a more pronounced callous-unemotional trait feature (i.e., low Agreeableness on the NEO-FFI), elevated sensation seeking, reward sensitivity, and alcohol and drug use, and more criminal and risk-taking behaviour. These features are consistent with fearlessness theory [89] as well as a “cold” antisocial phenotype [48]. Along these lines, cortisol exerts direct influence on the amygdala, with its outputs to the bed nuclei of the stria terminalis, the nucleus accumbens and the subgenual prefrontal cortex. Hence, reduced cortisol stress response may translate into a functionally weaker amydala. The neural circuit involving the amygdala-stria terminalis-medial regions of the prefrontal cortex has been implicated in the fear response; a hypo-functioning amygdala may lead to diminished anxiety and fear, and elevated arousal seeking, aggressive, impulsive, and substance misuse behaviours [90, 91]. In particular, the bed nuclei of stria terminalis and nucleus accumbens have links with the medial prefrontal cortex, the area where intuitive and affective inputs (i.e., somatic markers) may influence responses to emotional as well as social stimuli [74]. Dysfunction (e.g., lesions) in this medial prefrontal region has been linked to inadequate responses to social stimuli, reduced empathy, and the inability to abide by rules and social norms. In sum, disruption of this frontal-limbic juncture could lead to a kind of “hypofrontality” that would explain group MIXED’s markedly asocial disposition.

Our main analytic strategy did not directly reveal significant group differences in the degree of reduced cortisol stress response between the RD groups. Nevertheless, ancillary sensitivity analysis revealed more extreme blunting of the cortisol stress response in group MIXED when another substance-related risky behaviour, nicotine intake, was accounted for. Suggestively, group MIXED also showed greater drug misuse relative to the other RD groups. These findings are consistent with other research that found the strongest blunting of cortisol levels to be associated with drug use in addition to alcohol compared to alcohol misuse alone [92]. This effect is posited to reflect the added adverse impact drug use exerts on the forebrain and limbic structures outlined above [93]. Concern for drugged driving is growing in the traffic safety field [94], with some research indicating the highest recidivism rates in DWI drivers who misuse both alcohol and drugs [95]. Future research with larger samples and the addition of heavily drug-involved DWI drivers would provide the power and specificity needed to clarify the potential neurobiological contributions to this added driving risk.

Group SPEED showed a distinct personality and behavioural profile characterized by sensation seeking, disinhibition, risky decision-making style, and heightened risk-taking behaviour, features similar to those reported in other correlational research into non-alcohol related RD [2, 32, 39, 40, 84]. Moreover, group SPEED’s blunted cortisol stress response and heightened RD behaviour in simulation is consistent with findings from a naturalistic study we conducted previously in young novice drivers [51] using onboard cameras and g-force sensors. Lower cortisol stress response was associated with lower than expected declines in rates of non-alcohol related crash and near-crash events over an 18-month period.

Taken together, group SPEED’s risk-taking profile is in line with the psychological and neurobiological mechanisms posited by stimulation-seeking theory. Blunted cortisol stress response may lower cortisol feedback and stimulation of dopamine release at the nucleus accumbens (i.e., a low baseline mesolimbic dopamine). This effect is posited to both reduce the experience of reward, and heighten feelings of prolonged dysphoria. The experience of strong sensations through thrill seeking and risk taking help stimulate dopamine release. Hence, risk-taking behaviour in the driving context may represent an attempt to reacquire hedonic homeostasis via stimulation of nucleus accumbens dopamine release [87]. Future imaging studies could examine the neural basis of this hypothesis, as well as hypotheses concerning the neurobiological underpinnings of the other patterns of RD behaviour posited above.

### Limitations

The strengths of this study include a rare multidimensional analytic perspective, which provides a high degree of convergent and discriminative evidence, and recruitment of representative and relatively well-matched RD samples. The study possesses notable limitations as well, however. Only male drivers were recruited, limiting generalization of the findings to female drivers. The study’s cross-sectional design is inadequate for strong causal inferences regarding the role of individual characteristics in specific risk-taking behaviours. Moreover, DWI laws, enforcement practices, conviction thresholds, and vehicle use patterns can differ significantly between jurisdictions. Thus, the generalizability of the results may be limited in jurisdictions where these factors diverge significantly from those in Quebec and Canada. Relatedly, we detected group differences in ethnic composition. As ethnic background can influence driving behaviour [80] and even traffic enforcement practices in some jurisdictions [96], we were obliged to use a *post hoc* statistical strategy to account for this potential confound. Finally, heterogeneity in the driving characteristics of our RD groups was found; in particular, some reckless driving and DWI events that were not documented in driving records (i.e., the basis of group member designation in this study) were self-reported. At the same time, arrests for traffic violations are rare relative to their frequency of occurrence [97]. Hence, in cases where minimal inclusion criteria for group inclusion were met by documented events, considerably more engagement in the RD behaviours distinguishing the present groups is likely.

### Implications

These results are preliminary and require further confirmation. Nevertheless, speculation about their clinical meaning could help steer future RD prevention research. While groups DWI and MIXED share some features, notably alcohol misuse and engagement in DWI, their differences appear clinically significant. Relative to the other RD profiles considered here, the profile exhibited by group DWI may be the most amenable to interventions that aim to augment recall of the negative consequences of DWI behaviour and pre-emptively decouple alcohol use from driving (e.g., Preventing Alcohol-Related Convictions Program (PARC) [98]). In the case of drivers with uncontrolled alcohol use disorder, however, specialized alcohol use disorder treatment seems unavoidable [11].

The characteristics unique to group MIXED (i.e., DWI behaviour coupled with asocial and criminal behaviour and mixed substance use pattern) differentiate them from more “pure” DWI offenders, both in terms of their prognosis [95] and possibly their selective response to intervention. Indeed, the features of drivers in group MIXED question the appropriateness of alcohol use interventions alone, or approaches that pivot on authoritarian, moral, and/or empathic arguments [99]. Interventions like motivational interviewing and contingency management have been shown to be beneficial for offenders like those in group MIXED [100–103], possibly by being well-equipped to the meet the therapeutic challenges they pose: motivational interviewing evokes advantages of change from the offender’s own perspective, while contingency management uses reward to encourage positive behavioural change in reward-sensitive individuals. Finally, the impulsive and stimulation seeking features characterizing group SPEED suggests that such drivers could preferentially benefit from a strategy that complements deterrence and technological restraint systems like speed limiters with opportunities for engaging in stimulating experiences in a safe environment.

In sum, the present findings suggest that the propensity for engaging in specific forms of RD behaviour represents a useful and accessible marker for multidimensional research to both disentangle the heterogeneity in the RD population, and develop more personalized approaches to prevention.
